# Modifying routine emergency medical team introductory training to a virtual storytelling (*talanoa*) format for Pacific island countries and areas

**DOI:** 10.5365/wpsar.2023.14.6.1037

**Published:** 2024-04-22

**Authors:** Anthony T Cook, Sean T Casey, Erin E Noste

**Affiliations:** a World Health Organization Regional Office for the Western Pacific, Manila, Philippines.; b Faculty of Medicine & Health, School of Population Health, University of New South Wales, Sydney, New South Wales, Australia.; c Department of Emergency Medicine, University of California San Diego, California, United States of America.

Many governments of Pacific island countries and areas (PICs) have committed to establishing rapidly deployable, fully self-sufficient national emergency medical teams (EMTs). The momentum for EMT development has expanded following recent health emergencies, such as tropical cyclones, measles outbreaks and the coronavirus disease (COVID-19) pandemic, among others. (*1*, *2*) However, for the first two and a half years of the COVID-19 pandemic, all PICs instituted some form of border restrictions, including extended border closures in some countries and areas that limited the ability to hold in-person EMT member training, which had been a core element of EMT development in the Pacific since the start of the initiative in the Pacific in 2017.

Despite border restrictions, Pacific EMTs continued to request support for team development and training as outbreaks and climate-related events in the disaster-prone Pacific continued to affect vulnerable populations. (*3*) During the first year of the COVID-19 pandemic, Category 5 cyclones Harold and Yasa hit both Fiji (*4*, *5*) and Vanuatu. (*6*) Given travel restrictions enforced in each country, these cyclones required national responses without the assistance of international EMTs. Prior to the COVID-19 pandemic, the Fiji Emergency Medical Assistance Team (FEMAT) and Vanuatu Medical Assistance Team (VANMAT) had been established with trained and equipped team members, following multiple in-person training workshops.

The need for local and national response capabilities, and the limited availability of international assistance during the first year of the COVID-19 pandemic, emphasized the need for ongoing national EMT development and training in the Pacific. To meet this need, the World Health Organization (WHO) proposed a series of remote, interactive, online EMT member training sessions to engage existing and future Pacific EMT members during the COVID-19 pandemic.

## Methods

From July to September 2021, WHO facilitated and hosted a weekly webinar series to familiarize existing and potential EMT members across the Pacific with the essential concepts, principles and standards of the EMT initiative. These online sessions were 60–90 minutes long, with 45–60 minutes of presentation on specific topics (**Table 1**) and up to 30 minutes of open dialogue based on the Pacific tradition of storytelling or *talanoa*. In many Pacific languages, *talanoa* means to tell a story or have a conversation. (*7*) The webinar series content followed the principles of the *Classification and minimum standards for emergency medical teams* (2021), known as the “Blue Book,” (*8*) with specific themes from and for Pacific contexts.

Webinar sessions were divided into the following three themes: overview of the EMT initiative and coordination; EMT operational logistics and water, sanitation and hygiene (WASH); and EMT clinical care. The overview sessions included discussions on how the EMT standards and principles could be implemented in the Pacific in response to disasters and outbreaks, as well as on the EMT life cycle and how Pacific EMTs have operated in past emergencies. The first session under this theme stressed the importance of nationally-led coordination of both national and international EMTs. (*9*)

The webinar series included four EMT operational logistics and WASH sessions. The first provided an overview of EMT logistics focusing on how to deploy teams on small boats or aircraft that are most likely to be used in the Pacific. The next session outlined safety, security and communication in the field. The last operational logistics session provided a guide to EMT camp planning and setup, with a focus on deploying mobile EMTs to Pacific communities, villages and islands.

The remainder of the webinar sessions focused on EMT clinical service delivery, which included four separate sessions. The first provided an overview of clinical operations that an EMT in the Pacific is expected to perform, with experiences shared by EMT colleagues from Australia and Tonga. The second session provided details on how to plan an EMT’s pharmaceutical cache to deliver mobile medical care in remote or disaster-affected islands or areas. The third session covered team members’ physical and mental health, and the final session highlighted the role of EMTs in outbreak response based on many experiences in the Pacific, including COVID-19 response efforts in Fiji and the 2019 measles response in Samoa. The 11-week webinar series was designed to deliver a broad-based overview of EMT action in the Pacific.

Feedback was sought after every session via a questionnaire designed to inform changes for future sessions. The questionnaire addressed such questions as whether the session had covered all of its objectives, whether the images and text used during the session were clear and visible, whether the webinar had increased the participants’ understanding of the topics, whether participants would recommend this webinar to their colleagues, and if they had any suggestions on how to improve the sessions.

## Results

Over 300 participants from 23 countries and areas across the Pacific and other countries across the world participated in the 11 online webinar sessions. The average number of participants per session was 85. Individuals who could not attend a specific session were able to view the recorded presentations on a shared server or a dedicated YouTube link. (*10*) All presentations and resources were shared with the participants after the session through a Google Drive link.

Much of the feedback was positive, with a few suggestions on increasing the time dedicated to the Pacific tradition of *talanoa* and asking participants to share their experiences. The most significant adaptation of the sessions based on the feedback was the incorporation of the Pacific tradition of *talanoa*. This was included following feedback from the first session, with 30 minutes in each subsequent session dedicated to *talanoa*. These *talanoa* sessions enabled experienced faculty from EMTs in Australia, Cook Islands, Fiji, New Zealand, Papua New Guinea, Solomon Islands, Tonga and Vanuatu to share their Pacific deployment experiences through storytelling.

## Discussion

Adapting the webinar session plans and content to incorporate the *talanoa* style of communication in the Pacific facilitated an environment of learning from peers and colleagues. This sharing of stories increased attendee engagement in the online virtual setting. The Pacific EMT webinar series provided knowledge on the core standards, principles and modes of EMT operations, and increased engagement in the establishment and continued growth of the EMT initiative in the Pacific.
